# Effective tumor cell abrogation via Venetoclax-mediated BCL-2 inhibition in *KMT2A*-rearranged acute B-lymphoblastic leukemia

**DOI:** 10.1038/s41420-022-01093-3

**Published:** 2022-07-01

**Authors:** Anna Richter, Sandra Lange, Clemens Holz, Luisa Brock, Thomas Freitag, Anett Sekora, Gudrun Knuebel, Saskia Krohn, Rico Schwarz, Burkhard Hinz, Hugo Murua Escobar, Christian Junghanss

**Affiliations:** 1 Department of Medicine, Clinic III - Hematology, Oncology, Palliative Medicine, Rostock University Medical Center, Ernst-Heydemann-Str. 6, 18057 Rostock, Germany; 2 Institute of Pharmacology and Toxicology, Rostock University Medical Center, Schillingallee 70, 18057 Rostock, Germany

**Keywords:** Acute lymphocytic leukaemia, Preclinical research

## Abstract

Dysregulation of the intrinsic BCL-2 pathway-mediated apoptosis cascade is a common feature of hematological malignancies including acute B-lymphoblastic leukemia (B-ALL). The *KMT2A*-rearranged high-risk cytogenetic subtype is characterized by high expression of antiapoptotic protein BCL-2, likely due to the direct activating binding of *KMT2A* fusion proteins to the *BCL2* gene. The BCL-2 inhibitor venetoclax (VEN) has proven great clinical value in other blood cancers, however, data on B-ALL is sparse and past studies have not so far described the effects of VEN on gene and protein expression profiles. Using cell lines and patient-derived in vivo xenograft models, we show BCL-2 pathway-mediated apoptosis induction and decelerated tumor cell counts in *KMT2A*-rearranged B-ALL but not in other cytogenetic subtypes. VEN treatment of cell line- and patient-derived xenografts reduced blast frequencies in blood, bone marrow, and spleen, and tumor cell doubling times were increased. Growth rates are further correlated with VEN concentrations in blood. In vitro incubation with VEN resulted in BCL-2 dephosphorylation and targeted panel RNA sequencing revealed reduced gene expression of antiapoptotic pathway members *BCL2*, *MCL1*, and *BCL2L1* (BCL-XL). Reinforced translocation of BAX proteins towards mitochondria induced caspase activation and cell death commitment. Prolonged VEN application led to upregulation of antiapoptotic proteins BCL-2, MCL-1, and BCL-XL. Interestingly, the extrinsic apoptosis pathway was strongly modulated in SEM cells in response to VEN. Gene expression of members of the tumor necrosis factor signaling cascade was increased, resulting in canonical NF-kB signaling. This possibly suggests a previously undescribed mechanism of BCL-2-independent and NF-kB-mediated upregulation of MCL-1 and BCL-XL. In summary, we herein prove that VEN is a potent option to suppress tumor cells in *KMT2A*-rearranged B-ALL in vitro and in vivo. Possible evasion mechanisms, however, must be considered in subsequent studies.

## Introduction

Evasion of apoptotic processes is a hallmark of cancer and is frequently observed in several subtypes of hematological neoplasms [1, 2]. Extrinsic apoptosis is usually initiated outside the cell via tumor necrosis factor (TNF), Fas ligand, or TNF ligand (TRAIL)-induced death receptor activation and subsequent caspase 8 and 10 cleavage [3] or nuclear factor kappa B (NF-kB) activation, resulting in transcription of cell survival genes [4]. Intrinsic BCL-2-mediated apoptotic signaling features distinct pro- and antiapoptotic acting proteins. The signaling cascade can be initiated by stimuli like cellular stress, nutrient deprivation, or DNA damage, resulting in the activation of proapoptotic molecules BAD, NOXA, BIM, and BID. Those proteins inhibit the pro-survival proteins BCL-2, MCL-1, and BCL-XL. Blockade of the antiapoptotic proteins including BCL-2 disbands the inhibition of proapoptotic effector molecules BAX and BAK, leading to the formation of pores within the outer mitochondrial membrane. The resulting collapse of the membrane potential triggers cytochrome c release, caspase activation, and DNA fragmentation [2].

Increased BCL-2 activity, as well as dysregulation of further members of the BCL-2 signaling cascade, has previously been shown in acute lymphoblastic leukemia (ALL) and other hematological malignancies like acute myeloid leukemia (AML), chronic lymphoblastic leukemia (CLL), chronic myeloid leukemia, several lymphomas, or myeloma [2, 5]. Therapeutic intervention of this aberrantly regulated pathway using BCL-2 inhibitor venetoclax (VEN; ABT-199) thus seems beneficial and demonstrated clinical efficacy in CLL [6, 7] and AML patients [8, 9]. Early clinical trials are currently investigating the therapeutic potential and chemo-sensitizing properties of VEN in combination setups in adult ALL patients, with one study in T-ALL demonstrating encouraging results [10].

Like CLL and AML cells, ALL blasts are also often characterized by BCL-2 and BCL-XL over-expression, suggesting that VEN could also possess anti-leukemic potential in this entity. ALL patients with *KMT2A::AFF1* translocations (t(4;11), formerly known as *MLL::AF4*) have a particularly grim prognosis compared to other subgroups [11]. This cytogenetic subtype is characterized by high BCL-2 gene and protein expression profiles, possibly due to the direct binding of the fusion protein to the *BCL2* genetic site [5]. So far, there are no targeted therapies for *KMT2A::AFF1* translocated leukemias available. Occasional preclinical studies have investigated VEN in B- and T-ALL cell lines as well as primary samples, demonstrating encouraging results also in the high-risk subgroups of *KMT2A* and *BCR::ABL1*-rearranged B-ALL [12–19]. Those experimental series mainly correlated the basal expression of pro- and antiapoptotic proteins and VEN response but lacked detailed experimental setups to determine the exact mechanisms induced by VEN within the tumor cells.

The present study, therefore, aimed to characterize the VEN-induced effects on the cellular and molecular level in adult B-ALL with and without *KMT2A* rearrangements. Focusing on protein and gene expression patterns, we herein describe the response to VEN in vitro and in vivo and further elucidate a previously unknown evasion strategy of B-ALL cells.

## Results

### VEN reduces tumor cell counts in *KMT2A::AFF1*-rearranged B-ALL

**Fig. 1:**
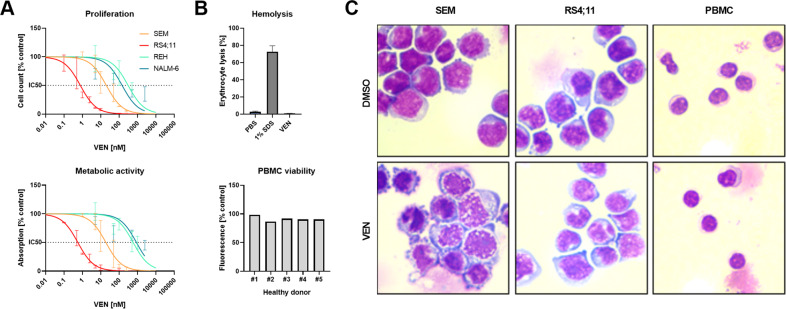
Influence of VEN on healthy cells and leukemic blasts. **A** Dose-response curves (nonlinear regression, curve fit) of cell lines SEM, RS4;11, REH, and NALM-6 were incubated with increasing concentrations of VEN for 72 h. Proliferation and metabolic activity were assessed by trypan blue staining and WST-1 assay, respectively. Mean ± SD of 1–3 biological replicates. **B** Erythrocyte and PBMC cytotoxicity were evaluated by hemolysis and calcein-AM assay, respectively. The blood of five healthy donors was used. Mean ± SD of three technical replicates. Hemolytic activity was assessed by hemoglobin release after 120 min incubation with 10 nM VEN or 1% SDS (positive control). For viability testing, PBMCs were incubated with 10 nM VEN or DMSO (control) for 24 h. **C** Cells were treated with 2.5 nM (RS4;11) or 10 nM (SEM, PBMCs) VEN for 48 h and subsequently spun onto microscopic slides and Pappenheim stained. Representative images of three independent biological replicates at 100x magnification.

To estimate the antiproliferative efficacy of VEN in B-ALL cells, we first incubated cell lines with *KMT2A::AFF1* translocation (SEM, RS4;11) and *KMT2A* wild-type cell lines REH, NALM-6 with increasing concentrations of VEN. All cell lines exhibit stable basal BCL-2 protein expression and activating phosphorylation of serine-70 (Fig. S1). VEN induced decreased tumor cell counts and metabolic activity in SEM and RS4;11 in low nanomolar concentrations, resulting in IC50 values of 20 nM (SEM) and <1 nM (RS4;11) (Fig. 1A and Table S2). *KMT2A* wild-type cell lines REH and NALM-6 demonstrated intrinsic resistance with IC50 values >500 nM. Concerning toxic potential on non-neoplastic healthy blood cells, no hemolysis, decreased PBMC viability (Fig. 1B) or morphological changes (Fig. 1C) were observed after VEN incubation. In contrast, SEM and RS4;11 cells showed severe signs of apoptotic processes, including increased cell size, membrane blebbing, disintegrated cell membranes, irregular and dissolving nuclei, or heavy vacuolization (Fig. 1C).

### VEN induces intrinsic apoptosis via BCL-2 dephosphorylation and subsequent Bax-mediated caspase activation

**Fig. 2:**
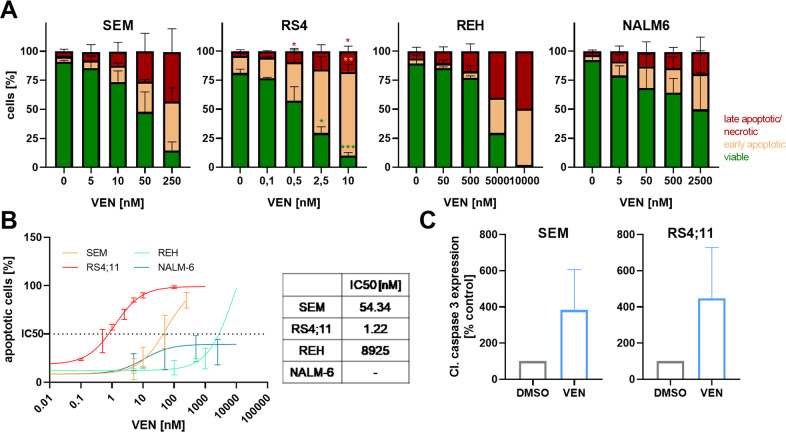
Influence of VEN on apoptosis induction. **A** Cells were incubated with increasing concentrations of VEN for 72 h before staining with Annexin V-FITC and propidium iodide and subsequent flow cytometry. Mean ± SD of 1–4 biological replicates; two-way ANOVA with post hoc Dunnett’s multiple comparisons test. Asterisks indicate significance compared to the respective DMSO control. **B** Dose-response curves (nonlinear regression, curve fit) of cell lines incubated with increasing concentrations of VEN for 72 h. Apoptotic cells were assessed by AnnV/PI staining and flow cytometry and early and late apoptotic/necrotic) cells were added to calculate the amount of apoptotic cells. Mean ± SD of 1–4 biological replicates. **C** Protein expression of cleaved caspase-3 was analyzed by intracellular flow cytometry after 48 h incubation with 2.5 nM (RS4;11) or 10 nM (SEM) VEN or DMSO (control). Mean ± SD of three biological replicates; paired *t*-test.

As morphology analyses indicated apoptosis induction, we next performed flow cytometry-based detection of early and late apoptotic processes. Reduced viable cell counts were observed in all cell lines (Fig. 2A); however, concentrations necessary to achieve apoptosis in half of the cells differed significantly, matching IC50 concentrations of cell count analyses and metabolic activity (Fig. 2B). VEN incubation induced signs of both, early and late apoptosis. Early apoptosis was detected most frequently in RS4;11 cells, which were also most responsive to apoptosis induction overall. Analyzing downstream signaling cascades, we observed a fourfold increase in effector caspase-3 cleavage in SEM and RS4;11 cells (Fig. 2C).

**Fig. 3:**
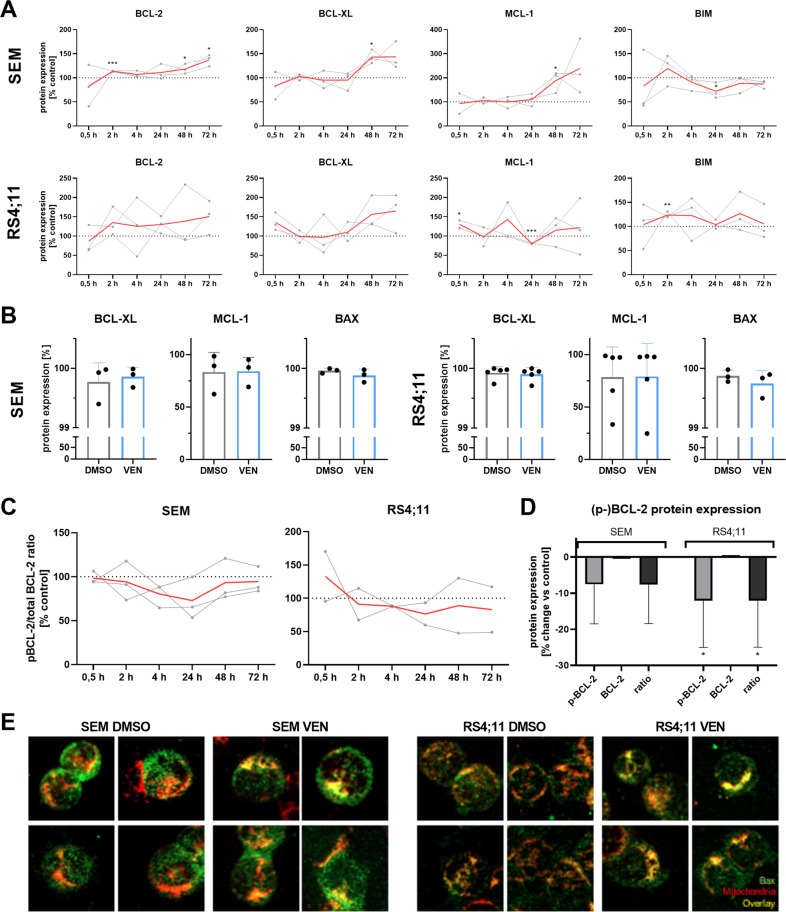
Effect of VEN on BCL-2 pathway-mediated apoptotic signaling. **A** Expression of BCL-2 pathway proteins was assessed by immunoblot. Two to three individual biological replicates (gray) and expression mean (red); multiple *t*-tests, asterisks indicate significance vs time-matched DMSO control. **B** Total BCL-2 pathway member protein expression was analyzed by intracellular flow cytometry after 48 h incubation. Mean ± SD of three to five biological replicates; ratio paired *t*-test. **C** The time-dependent influence of VEN on BCL-2 phosphorylation was measured by immunoblot. BCL-2 and p-BCL-2 bands of three (SEM) or two (RS4;11) individual biological replicates were quantified. Relative BCL-2 phosphorylation values of all replicates (gray), as well as the mean of those experiments (red), are indicated in the graphs. Ratio paired *t*-test. **D** Protein expression of phosphorylated and total BCL-2 was assessed by intracellular flow cytometry after 48 h incubation with VEN or DMSO (control). Absolute expression values of controls was set to 100% and the relative change in protein expression following VEN incubation is indicated in the figure. Mean ± SD of five (SEM) or four (RS4;11) biological replicates; paired *t*-test of each protein vs. respective control. **E** Functional assessment of Bax-mediated apoptosis induction was performed by Bax translocation assay after 48 h VEN incubation. Four representative images of four biological replicates per cell line and treatment group, 40-fold magnification.

Following we investigated if apoptosis was induced via specific modulation of the BCL-2-mediated intrinsic apoptosis pathway. First, we investigated the protein expression of antiapoptotic proteins BCL-2, BCL-XL, and MCL-1 as well as proapoptotic pathway members BIM and BAX (Fig. 3A, B). Interestingly, the expression of antiapoptotic proteins increased in response to prolonged VEN application (>48 h) in immunoblot analyses, indicating a rise of protein molecules within the samples (Fig. 3A). Shorter incubation periods did not result in any changes. Quantifying the number of cells expressing the respective proteins by intracellular flow cytometry (Fig. 3B), no such changes were detected. This suggests that cells with basal antiapoptotic protein expression increased the amount of BCL-2, MCL-1, and BCL-XL molecules in response to prolonged VEN treatment while negative cells remained as such. In contrast, apoptotic effector proteins BAX and BIM remained largely unaffected in both, immunoblotting and flow cytometric experiments.

Investigating the active form of BCL-2, we found that VEN induced dephosphorylation and thus inactivation of BCL-2 during early incubation time points in both, immunoblot and intracellular flow experiments (Fig. 3C, D). Extended treatment time frames did not result in further modulation of the phosphorylated-to-total protein ratio. Those effects were observed in both cell lines.

In order to investigate the BCL-2 downstream signaling cascade to evaluate whether VEN-induced effects are pathway-specific, we performed BAX translocation assays and immunofluorescence imaging to show the spatial overlap of the BAX effector protein and mitochondria, where BAX induces pore-like structures and thus initiates apoptosis induction (Fig. 3E). Following VEN incubation, BAX translocated from the cytoplasm towards mitochondria in both cell lines investigated, indicated by a yellow overlap signal of BAX molecules (green) and mitochondria (red), resulting in apoptosis commitment.

### Low-dose VEN differentially regulates apoptosis and leukemia-related genes in SEM and RS4;11 cells

**Fig. 4:**
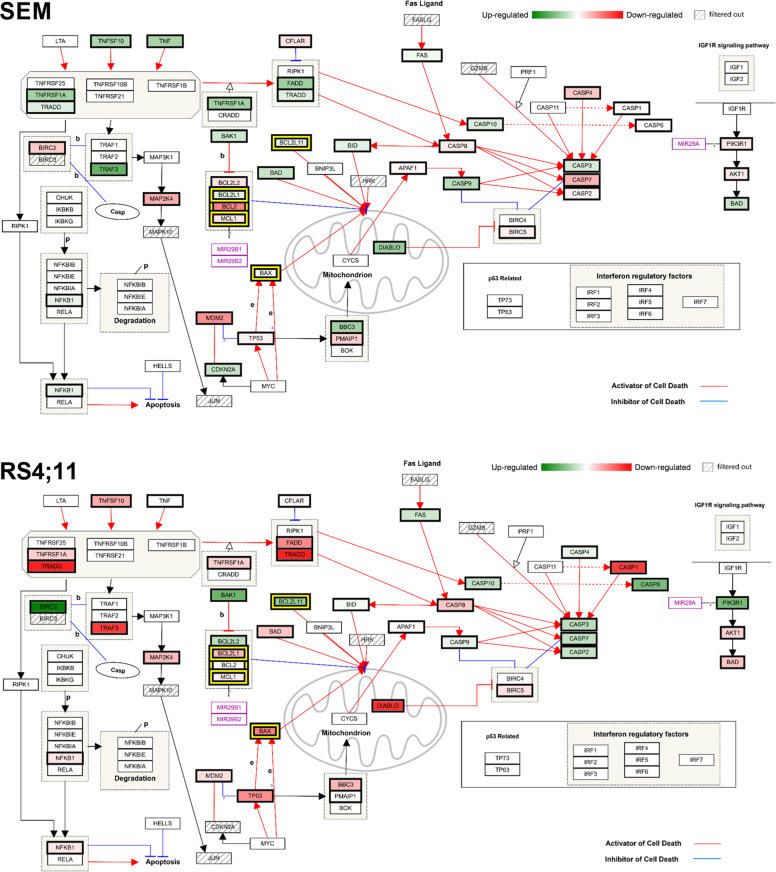
Effect of VEN incubation on gene expression of apoptotic signaling cascades in SEM (upper panel) and RS4;11 cells (lower panel). Gene expression was assessed by targeted RNA sequencing and analyzed using Transcriptome Analysis Console 4.0 software. The WikiPathways plugin was applied to generate pathway maps of genes involved in apoptotic signaling. Green and red colored gene boxes indicate up- and down-regulation following VEN incubation, respectively and darker shades symbolize stronger effects. Genes with average of less than 50 total reads were excluded to justify biological significance (marked as filtered out).

We next investigated whether VEN application induced gene expression changes in apoptosis and leukemia-related genes in SEM in RS4;11 cells. The basal expression in both cell lines was rather similar (Fig. S2) but with some genes within the BCL-2 signaling pathway differentially transcribed (Fig. S2A, D, E left panel). Incubation with VEN usually induced up- or down-regulation of a respective gene in both cell lines; however, while the expression of the antiapoptotic protein BFL1 (*BCL2A1* gene) was downregulated in SEM, it was higher expressed after treatment in RS4;11. The opposite was observed for apoptosis-inducer *BAD*, which was upregulated in SEM but downregulated in RS4;11 (Fig. S2E, right panel). The gene expression of BCL-2 pathway genes was generally rather reduced after VEN incubation in both cell lines (Fig. 4 and Figs. S3, S4) irrespective of the gene’s functions. In SEM, upregulation occurred in proapoptotic genes *BAD* and *BAK1* while the inhibitors of apoptosis *BCL2* and *BCL2A1* demonstrated the broadest downregulation (Fig. S3C). Similar effects were observed in RS4;11, except for a strong upregulation of antiapoptotic protein BFL1 (*BCL2A1*), which had, however, a very low basal expression. Proapoptotic effector proteins BIM (*BCL2L11*) and BAK (*BAK1*) were further upregulated (Fig. S4C).

Mapping gene expression patterns and the WikiPathways Apoptosis cascade [20], we observed that not only BCL-2 pathway members were regulated by VEN. Extrinsic apoptotic cascades were also affected in both cell lines (Fig. 4, upper left part of the graphs). Interestingly, gene regulation was completely opposite in SEM and RS4;11, with the entire TNF pathway including TNF receptor (*TNFRSF1A*), TNF ligand TRAIL (*TNFSF10*), TNF receptor-associated proteins (*TRADD*, *TRAF3*, and *FADD*) and effector molecule *NFKB1* upregulated in SEM but inhibited in RS4;11.

Considering the entire gene panel of over 200 molecules involved in apoptosis and leukemogenesis, *BCL2* was among the most downregulated genes in SEM (Fig. S3), together with tumor drivers and other inhibitors of apoptosis-like *HOXA3, MDM2*, and *VDAC2*. However, also prominent tumor suppressors like *PTEN* and *FOXO1* as well as apoptosis-inducers *PTRH2* and *STK17B* were relevantly downregulated (Fig. S3B). On the other hand, the most upregulated genes were mainly apoptosis inducers like *TRAF3, DAPK3*, *FOXO4*, or *DDIT1*. The VEN-evoked effects in RS4;11 cells were rather different, with extrinsic apoptosis initiators *TRADD*, *TRAF1*, *STK3*, *CASP1*, and *ACIN1* being among the top downregulated genes (Fig. S4B). Both, proapoptotic BCL-2 or MAP kinase pathway members (*BIK*, *BAK1*, *MAP3K12*, and *MAPK9*) and proliferation-associated tumor drivers (*FOS*, *HOXA3*, and *POLR2B*) had an increased gene expression.

Finally, we performed quantitative RT PCR analyses of the central tumor and leukemia propagator as well as cell cycle regulator *MYC* in both cell lines, demonstrating a significant reduction in *MYC* mRNA after VEN treatment in RS4;11 but not SEM cells (Fig. S5A). *MYC* was not included in our RNA seq panel due to very high basal expression levels that would have resulted in diminished total read counts of all other genes. Further investigating if VEN incubation could therefore also influence cell cycle control, we evaluated the gene expression of *MYC* downstream target *CDK6* and the distribution of cell cycle phases. No changes were observed in either cell line (Fig. S5B, C).

### VEN treatment results in decelerated tumor cell proliferation in vivo

**Fig. 5:**
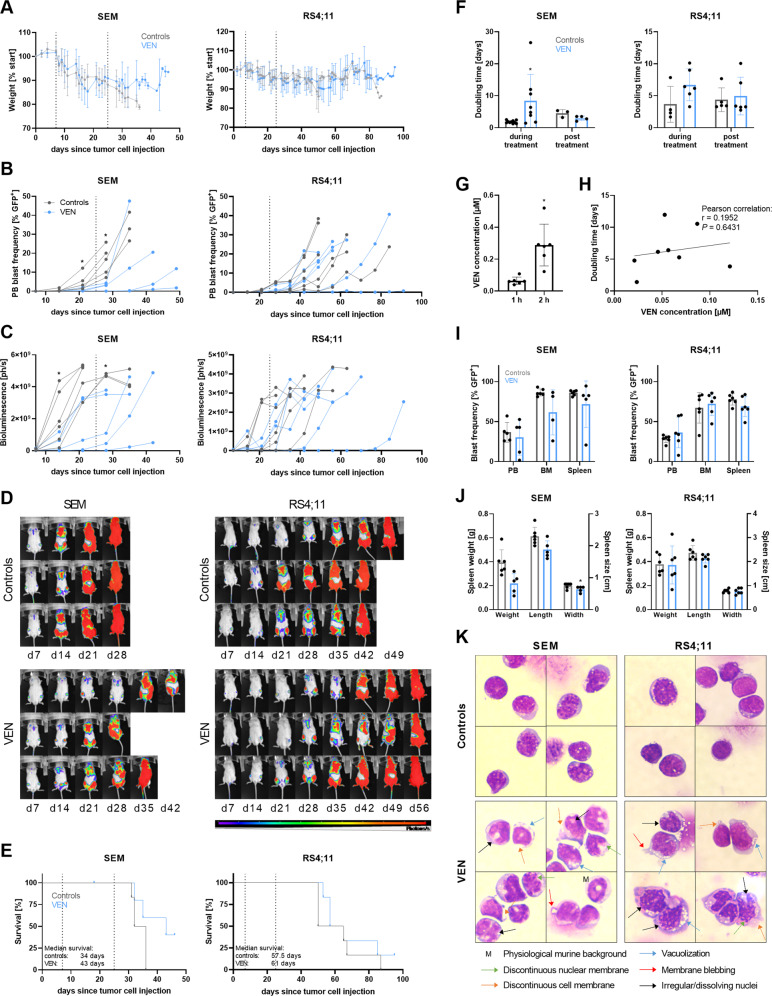
Effects of VEN treatment in cell line-derived xenograft models. **A** Weight progression of ten animals per study group. The dotted lines indicate the treatment period. Multiple *t*-tests. **B**, **C** Tumor cell proliferation was monitored by peripheral blood (PB) blast frequency measurement via flow cytometry (GFP + cells, **B**) and in vivo bioluminescence imaging (BLI, **C**). Each line represents an individual animal. BLI imaging was discontinued when technical saturation was reached. Mean ± SD of ten animals per group where four animals were sacrificed following therapy finalization at d25 (dotted line); Mann–Whitney test vs. time-matched controls. **D** Representative BLI images of three individual mice per group in ventral position. **E** Kaplan–Meier survival analysis. The dotted lines indicate the treatment period. Six animals per group; log-rank test. **F** The growth rate of leukemic blasts was calculated based on PB blast frequency values measured by flow cytometry. Values of d14 and d21 were used to assess the doubling time during treatment while values of d28 and d35 (SEM) or d35 and d42 (RS4;11) were used for posttreatment calculation. Mean ± SD of three to ten animals per group and time point; Mann–Whitney test. **G** Pharmacokinetic analyses were conducted one and 2 h after VEN p.o. application to investigate VEN concentrations in PB by liquid coupled mass spectrometry. Each dot represents an individual animal. Mean ± SD of six VEN-treated animals; Wilcoxon matched-pairs signed-rank test. **H** Analysis of VEN concentration in peripheral blood 24 h after application and the tumor cell doubling time of the respective animal during VEN treatment. Cumulative analysis of SEM and RS4;11-derived xenograft models. Linear regression where each dot represents an individual animal. Pearson’s correlation. **I**, **J** Determination of blast frequency in PB, bone marrow (BM), and spleen by flow cytometry (**I**) and spleen parameters (**J**) were assessed when the mice reached humane endpoints (30% blasts in PB or weak performance status). Mean ± SD of five to six animals per group; Mann–Whitney test. **K** Isolated BM or spleen cells were spun onto microscopic slides and Pappenheim stained. Four representative images of five to six mice per group at 100x magnification.

To investigate the effect of VEN on B-ALL cells in vivo, we applied two cell line (SEM, RS4;11)-based orthotopic xenograft models. The therapeutic procedure resulted in partly reversible weight loss in both models and controls as well as VEN-treated animals, probably due to the daily oral gavage process or substances included in the vehicle solution (Fig. 5A). The growth rate of tumor cells was assessed by weekly examination of blast frequencies in PB (Fig. 5B) and absolute tumor cell counts (Fig. 5C, D) using flow cytometry and longitudinal in vivo bioluminescence imaging, respectively. Tumor cell counts were significantly decelerated in the SEM-derived model whereas effects were less prominent in RS4;11 xenografts. The observed effects occurred mainly during the therapeutic time frame and tumor cell proliferation resumed after VEN cessation, resulting in mildly increased median survival periods (Fig. 5E). Calculation of tumor cell proliferation rates further confirms this effect, demonstrating significantly higher blast doubling times in VEN-treated SEM-derived mice compared to control animals (Fig. 5F). After therapy cessation, blast proliferation rates resembled those of the control animals. Similar values were measured for RS4;11-derived animals, albeit not significant.

Following we performed pharmacokinetic analyses to assess the achievable VEN concentrations as well as the duration of VEN persistence in PB (Fig. 5G). The highest concentrations were reached 2 h after p.o. VEN application. Severely lower concentrations were measured 24 and 72 h after the final VEN dosage, with no VEN detectable in most animals after 72 h. No traces of VEN were measured in animals treated with vehicle solution. Very high inter-animal variations in VEN concentrations led us to correlate the assessed VEN concentrations with the respective tumor cell doubling time of the individual animals (Fig. 5H). Mice with lower VEN concentrations in PB 24 h after the final VEN dose indeed had shorter blast doubling times, accounting for faster proliferation rates.

Upon experiment termination, bone marrow and spleen cells were isolated and tumor cell frequencies were determined by flow cytometry (Fig. 5I). No differences between controls and VEN-treated animals were observed in blood, bone marrow, and spleen blast frequencies of either xenograft model. Spleen size and weight were also unaffected (Fig. 5J). Blast morphology analyses, on the other hand, revealed prominent alterations in VEN-treated animals even several days to weeks after therapy cessation, while normal murine cells and blasts of control animals maintained their physiological appearance (Fig. 5K). Tumor cells appeared larger than in control animals and the cytoplasm-to-nucleus ratio increased. Similar to cell culture experiments (Fig. 1C), blasts demonstrated high vacuolization, discontinuous cell and nuclear membranes, irregular or dissolving nuclei, and membrane blebbing, all signs of apoptotic processes.

**Fig. 6:**
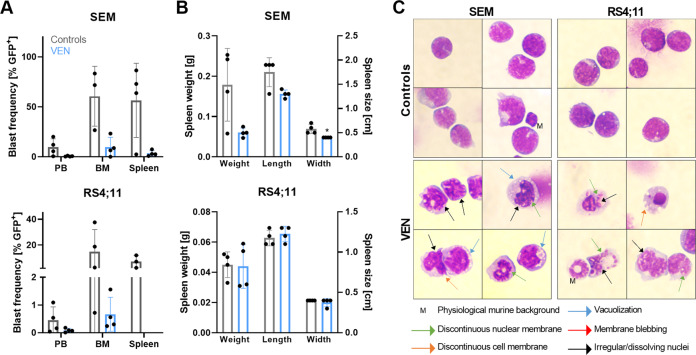
Immediate effects of VEN application on cell line-based xenograft models. Four mice per study group and model system were euthanized directly after therapy cessation. **A** Peripheral blood (PB), bone marrow (BM), and spleen blast frequencies were assessed by flow cytometry. No tumor cells were detected in spleens of RS4;11-derived xenograft mice after VEN therapy. Mean ± SD; Mann–Whitney test. **B** Spleens were weighed and measured immediately after experiment termination. Mean ± SD; Mann–Whitney test. **C** Isolated BM or spleen cells were spun onto microscopic slides and Pappenheim stained. Four representative images per xenograft model and study group at 100x magnification.

To evaluate whether the observed absence of alterations in the bone marrow and spleen blast frequencies was due to the long period between therapy cessation and experiment termination; and to assess the short-term effects of VEN treatment, we then performed a similar small in vivo approach as in the experiments above but mice were euthanized directly after the final VEN application. As expected, PB, bone marrow, and spleen blast frequencies were greatly lowered in VEN-treated mice compared to controls, albeit not significant probably due to low sample numbers or interindividual variance (Fig. 6A). Spleen weight and size were also reduced in the generally fast-proliferating SEM xenograft model while no changes occurred between treated and untreated RS4;11 animals (Fig. 6B). In the latter animals, spleens had the proportions of healthy, non-leukemic animals, probably due to the slow-proliferating nature of RS4;11 cells where the tumor burden at this time point was not sufficient to rise the spleen size and weight. Morphological analyses confirmed previous results and demonstrated severely altered and damaged blasts after VEN exposure in both models (Fig. 6C). This suggests that the observed reduced tumor cell frequency in bone marrow and spleen after VEN treatment is at least in part due to apoptosis and not only evoked by inhibition of proliferation.

Cell line-based model systems only resemble the clinical situation to a certain extent; therefore, six PDX models of adult ALL patients with *KMT2A* rearrangements (*KMT2A*-r) or *BCR::ABL1* translocation were selected and VEN-treated with the same schedule as the cell line-based models. All selected patients had stable BCL-2 protein expression comparable or higher than healthy donor PBMCs (Fig. S6). Once control animals reached the 30% blasts in PB threshold, all control and VEN-treated mice were euthanized to compare the induced effects.

**Fig. 7:**
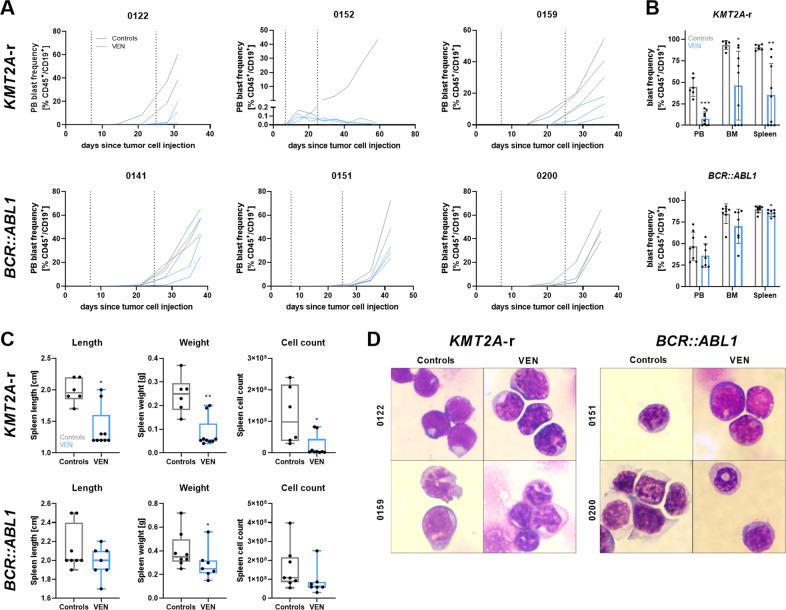
Effects of VEN treatment in PDX models. Analysis of one to three individual animals per PDX model and intervention group. **A** Tumor cell proliferation of *KMT2A* (upper panel) and *BCR::ABL1* rearranged models was monitored by peripheral blood (PB) blast frequency measurement via flow cytometry (CD45^+^/CD19^+^). Each line represents an individual animal. Dotted lines represent the start and end date of the treatment interval. **B** Determination of blast frequency in PB, bone marrow (BM), and spleen by flow cytometry after experiment termination. Control and VEN-treated mice of all *KMT2A* (upper graph) or *BCR::ABL1* rearranged models are summarized. Each dot represents an individual animal. Mean ± SD, unpaired *t*-test with Welch’s correction or Mann–Whitney test. **C** Box plots (min/max) of spleen length, weight, and cell count of *KMT2A* (upper panel) and *BCR::ABL1* rearranged models. Controls and VEN-treated mice of all models per cytogenetic subtype are summarized. Each dot represents an individual animal. Mann–Whitney test. **D** Representative images of blast morphology of *KMT2A* (left panel) and *BCR::ABL1* rearranged PDX models treated with vehicle or VEN. Pappenheim staining of bone marrow cells. 100-fold magnification.

All *KMT2A*-r models demonstrated decelerated tumor cell counts during and following VEN treatment (Fig. 7A, upper panel). In line, blast frequencies in blood, bone marrow, and spleen upon experiment termination were significantly reduced (Fig. 7B, upper panel), as were spleen size, weight, and cell count (Fig. 7C, upper panel). The most intriguing results were achieved in the patient 0152-derived model, where no tumor cells could be detected in PB, bone marrow or spleen even five weeks after therapy cessation. Interestingly, and in sharp contrast to *KMT2A*-r models, BCL-2^+^ *BCR::ABL1* mutated samples did not respond to VEN application, with similar blast kinetics and tumor cell frequencies in controls and treated animals (Fig. 7A, B, lower panels). However, spleen parameters were slightly reduced following VEN treatment (Fig. 7C).

Morphological analyses revealed distinct patterns in the individual PDX models (Fig. 7D). Patient 0122-derived blasts had a more homogenous nuclear staining than the other models while tumor cells of patient 0159 and 0200-engrafted mice were bigger than those of the other patients. Blast morphology after VEN treatment resembled the cell line-derived models, albeit not as severely as previously seen. Irregular nuclei and an increased cytoplasm-to-nucleus ratio were observed especially in *KMT2A*-r models. Matching the growth and engraftment data, *BCR::ABL1*-translocated blasts were only mildly affected by VEN incubation and demonstrated little to no morphological changes, except for an increase in cytoplasmic volume in patient 0151-derived cells.

## Discussion

Dysregulated BCL-2 signaling is a hallmark of many hematological neoplasms, including B-ALL [2]. The selective BCL-2 inhibitor VEN has proven great clinical activity in CLL [6, 7] and AML [8, 9], offering deep and durable remissions in a subgroup of patients. Although some preclinical studies [5, 13–16, 18, 19] and early case reports [10, 21] have evaluated the effect of VEN in ALL, hardly any data exist for the subgroup of *KMT2A*-r B-ALL. We herein demonstrate that BCL-2 inhibition is a promising method to induce tumor cell abrogation in this high-risk cohort.

VEN in vitro incubation of *KMT2A*-r cell lines SEM and RS4;11 with low nanomolar and physiologically achievable concentrations resulted in severely reduced tumor cell counts, apoptosis induction, and morphological changes. Other groups conducting basic viability screenings with the same cell lines achieved comparable results [5, 14, 22, 23]. In vivo application of VEN induced decelerated tumor cell growth rates and reduced blast frequencies in bone marrow and spleen in both *KMT2A*-r cell line-derived xenograft systems. Gauert et al. used SEM cells in a zebrafish xenograft model and found comparable results [23]. The fact that mice engrafted with RS4;11 cells, which were highly responsive to VEN incubation in vitro, seemed to benefit less from VEN therapy than SEM-derived models, is most likely due to the faster proliferation kinetics of SEM cells compared to RS4;11. Analyzing tumor cell frequencies directly after therapy cessation demonstrated high VEN efficacy also in RS4;11 xenografts, proving that this model is responsive, too. Prolonged application periods or delayed therapy initiation would probably increase the therapeutic efficacy in RS4;11-derived xenografts.

We used three PDX model systems of adult *KMT2A*-r samples to further validate the promising results of VEN treatment in B-ALL. All models responded well, matching the findings of other groups who reported high VEN efficacy in *KMT2A*-r PDX models [5, 13, 14, 24]. We subsequently also evaluated the effect of VEN on other B-ALL cytogenetic subtypes but could not detect any anti-leukemic effects in *BCR::ABL1* positive PDX models. This is in line with the observations of others who also evaluated the VEN efficacy in different molecular subgroups, describing little to no potential in patients with *BCR::ABL1* translocations or other cytogenetic aberrations [5, 15, 24]. A possible explanation for this phenomenon is the fact that the *KMT2A::AFF1* fusion gene directly binds *BCL2* [25, 26] and thus regulates its gene expression, resulting in and maintaining high basal protein levels via histone H3 lysine 79 di- and trimethylation [5].

In line with another study in CLL patients [27], we observed a mild over-expression of antiapoptotic proteins MCL-1 and BCL-XL in response to prolonged VEN incubation. This might be explained by compensatory mechanisms of the cells and has also been discussed as a potential resistance mechanism to VEN treatment [27]. We thus designed a panel for targeted RNA sequencing of apoptosis and leukemia-relevant genes to elucidate potential evading strategies initiated by VEN-incubated B-ALL cells.

In SEM cells, we detected upregulated TNF signaling, resulting in increased canonical NF-kB activity following VEN incubation. In mantle cell lymphoma cells it was shown that VEN sensitivity was impaired via NF-kB pathway upregulation, leading to over-expression of MCL-1 and BCL-XL and thus mediating VEN resistance [28]. Similar observations were described in CLL, where VEN resistance was induced by CD40-induced upregulation of antiapoptotic proteins [29] via both, noncanonical and canonical NF-kB signaling [30, 31]. In CLL, CD40 is frequently over-activated in response to immunomodulatory effects, demonstrating that the tumor microenvironment can play a significant role in VEN response and resistance and offers possibilities for therapeutic intervention [30, 32, 33]. We herein show for the first time that canonical NF-kB signaling and subsequent upregulation of antiapoptotic proteins in response to VEN can be activated via the TNF pathway and without microenvironment-mediated CD40 expression as the effect was observed even without co-cultivation of microenvironment-mimicking stromal cells. However, further mechanistic studies need to be conducted to validate this hypothesis.

In summary, this study investigates the therapeutic potential of VEN in B-ALL in vitro and in vivo, demonstrating significant anti-leukemic efficacy in *KMT2A*-r cell lines and PDX models in a pilot study with limited sample numbers. Analyzing gene expression patterns of apoptosis and leukemia-related pathway members following VEN incubation, we revealed that TNF signaling, previously unrelated to VEN response, regulates apoptosis induction and thus possible VEN resistance in SEM cells. These results strongly argue for reinforced clinical investigation of VEN for *KMT2A*-r B-ALL, also in combination with immunomodulatory options.

## Materials and methods

A detailed description of all methods can be found in the online Supplementary Data.

### Cell lines and cultivation

Human B—ALL cell lines SEM, RS4;11, REH, and NALM-6 were purchased from DSMZ (Braunschweig, Germany) and maintained at 37 °C and 5% CO_2_ in IMDM medium (SEM), Alpha MEM medium (RS4;11) or RPMI 1640 medium (REH, NALM-6), all supplemented with 10% heat-inactivated fetal calf serum and 100 µg/ml penicillin/streptomycin (all PAN—biotech, Aidenbach, Germany). The medium was exchanged twice weekly and cells were seeded at a density of 3.3 × 10^5^ cells per ml for further culturing or inhibitor experiments. Cells were regularly checked for authenticity (cell surface flow cytometry) and mycoplasma contamination.

### Targeted RNA custom panel sequencing

SEM and RS4;11 cells were incubated with vehicle or 10 or 2.5 nM VEN, respectively, for 24 h before cells were harvested and washed twice in cold PBS. Subsequent RNA isolation and cDNA synthesis were performed using the RNeasy Mini Kit (Qiagen, Hilden, Germany) and SuperScript IV VILO Mastermix (Thermo Fisher Scientific, Waltham, MA, USA) according to the manufacturer's protocols. Gene expression analyses were carried out using the Ion GeneStudio S5™ Plus system. For targeted RNA sequencing, a custom panel was designed with the Ion AmpliSeq Designer, containing 214 genes associated with apoptosis, VEN response, or leukemic signaling (Table S1). Ion AmpliSeq RNA libraries were prepared according to the manufacturer’s protocol using Qubit RNA HS Assay Kit, Ion AmpliSeq Library Kit Plus, and Ion Library TaqMan Quantitation Kit. Following this, template preparation was carried out with the Ion 540 Kit-Chef using the Ion Chef instrument. The sequencing reaction run was performed with the Ion GeneStudio S5™ Plus system and 500 flows. The evaluation of datasets was performed using Transcriptome Analysis Console (TAC) Software 4.0.2.15 (Thermo Fisher Scientific). Genes with total reads average below 50 were filtered out to focus on biologically relevant changes. Individual biological replicates were considered as repeated measurements. Four and three biological replicates were analyzed for SEM and RS4;11 cells, respectively.

### In vivo model systems

All animal experiments were approved by the review board of the federal state Mecklenburg-Vorpommern, Germany (reference number: LALLF MV/7221.3-1.1-063/20). Eight to twelve weeks old male and female NOD.Cg-*Prkdc*^*scid*^*Il2rg*^*tm1Wjl*^/Szj (NSG) mice were bred and housed in the accredited laboratory animal Core Facility of the Rostock University Medical Center with access to water and standard chow ad libitum. All experiments were carried out in a laboratory setting and no intervention was performed within the animal housing and breeding rooms. SEM and RS4;11 cells lentivirally transduced with GFP and luciferase (SEM-fluc, RS4;11-fluc) [34], or primary adult ALL cells amplified in a patient-derived xenograft (PDX) model system were used for inhibitory experiments. Primary cells were isolated as previously described [35]. The study was performed in accordance with the Declaration of Helsinki and the local ethical standards of the Rostock University Medical Center. All participants gave informed consent. Tumor cell injection, monitoring of blast counts, and distribution using in vivo bioluminescence imaging (BLI) and peripheral blood (PB) flow cytometry (GFP^+^ or CD45^+^/CD19^+^) were performed as previously described [35–38].

### In vivo treatment procedures and study endpoints

For cell line-derived xenograft model systems (SEM-fluc, RS4;11-fluc), ten animals each were treated with either VEN or vehicle on five days of three consecutive weeks starting 7 days after tumor cell injection. Group sizes were calculated in G*power based on expected effect sizes, a statistical power of 0.8, and an alpha error of 0.05 for cell line-derived xenograft models. Only mice with a bioluminescence signal indicating successful tumor cell engraftment were included in experiments. Randomization was performed based on sex, age, weight, and quantitative BLI signal on day 7 after cell injection. Study groups were not blinded to the investigators. VEN was dissolved in 60% Phosal 50 PG (Lipoid, Ludwigshafen, Germany), 30% PEG400 (Carl Roth, Karlsruhe, Germany), and 10% ethanol and applied via oral gavage. During the first week of treatment, VEN concentrations were increased according to the clinically applied protocol, starting at 20 mg/kg body weight, and raised daily by 20 mg/kg until day 5 of treatment, reaching the maximum dose of 100 mg/kg body weight. Mice were monitored and weighed daily and BLI, as well as PB blast frequency measurement, was performed once per week as previously described [35–37]. Four mice per study group were euthanized by narcotization (75 mg/kg ketamine, 5 mg/kg xylazine) followed by cervical dislocation after the last VEN dose. The other six animals per group were regularly monitored until PB blast frequencies reached ≥30% or the mice met pre-defined humane endpoints. For PDX models, six mice per PDX model were injected with cells derived from one patient. Three mice each were treated with either VEN or vehicle as described above. Once one out of the six animals reached minimum of 30% blasts in PB, all six mice were euthanized.

### Statistical analyses

All values are expressed as mean ± standard deviation. Gaussian normality distribution was tested in all cases using Kolmogorov–Smirnov test, determining the following parametric or nonparametric with a post hoc test. The exact test is indicated in the respective figure legends. Kaplan–Meier curves and respective statistics were applied to estimate survival benefits. Statistical analyses were performed using GraphPad PRISM software (version 8). Statistical significance was defined as *P < 0.05, **P < 0.005, and ***P < 0.001.

## Supplementary information

Table S1

Table S2

Figure S1

Figure S2

Figure S3

Figure S4

Figure S5

Figure S6

Supplementary Figure Legends

Supplementary methods

Original Western Blots

List of antibodies

## Data Availability

The datasets generated during and/or analysed during the current study are available from the corresponding author on reasonable request.
