# Supplementary figures and images for: Effective tumor cell abrogation via Venetoclax-mediated BCL-2 inhibition in KMT2A-rearranged acute B-lymphoblastic leukemia
Source: Cell Death Discov. 2022 Jul 1;8:302. doi: 10.1038/s41420-022-01093-3 (PMC9249764; doi:10.1038/s41420-022-01093-3)

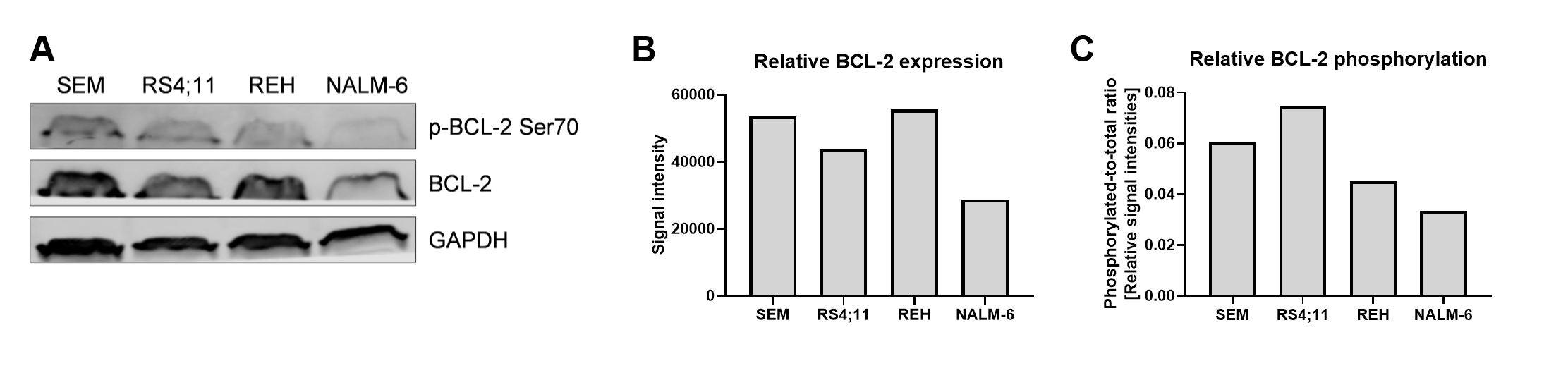

Supplement: Supplementary file 3 — Figure S1 [file 41420_2022_1093_MOESM3_ESM.png]

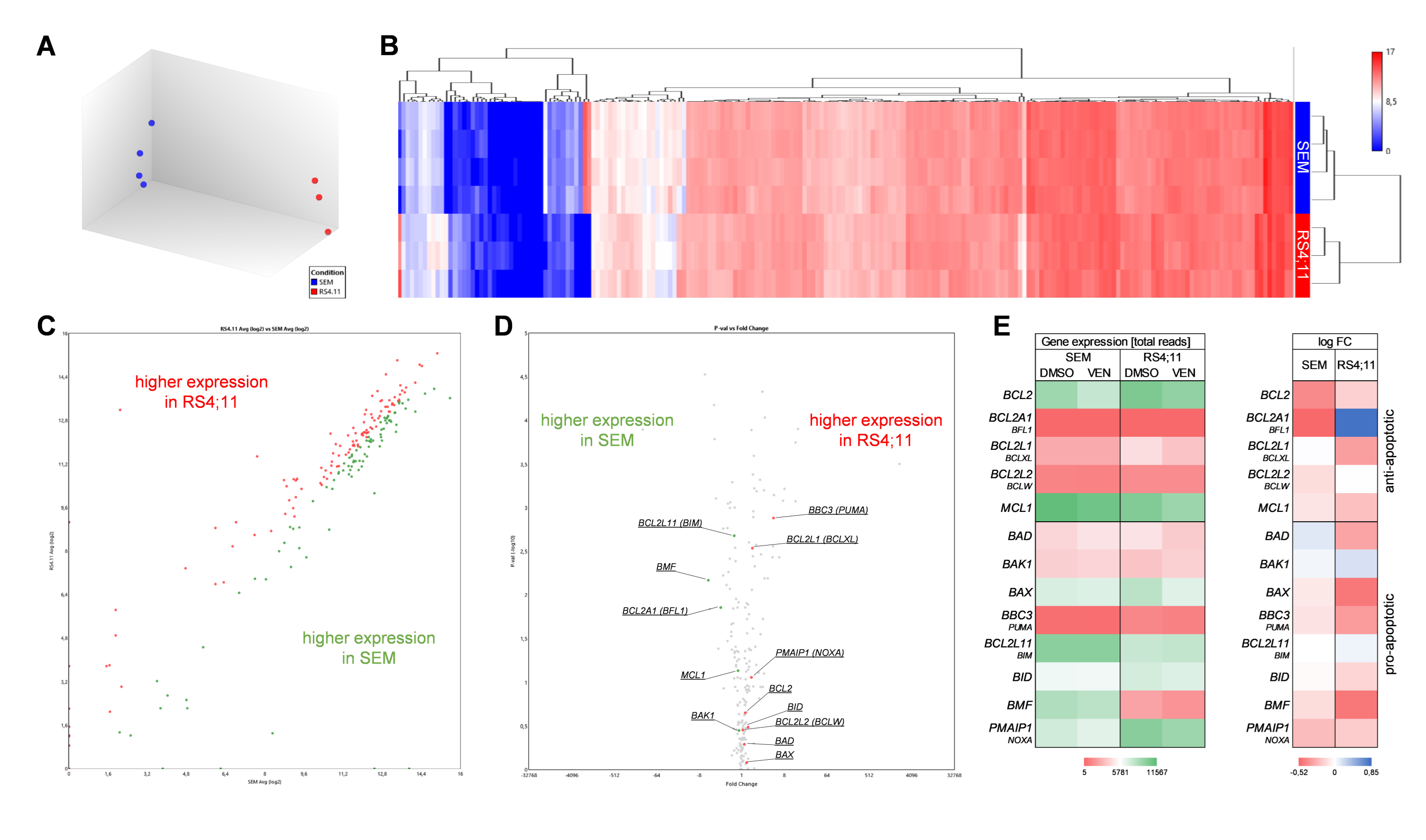

Supplement: Supplementary file 4 — Figure S2 [file 41420_2022_1093_MOESM4_ESM.png]

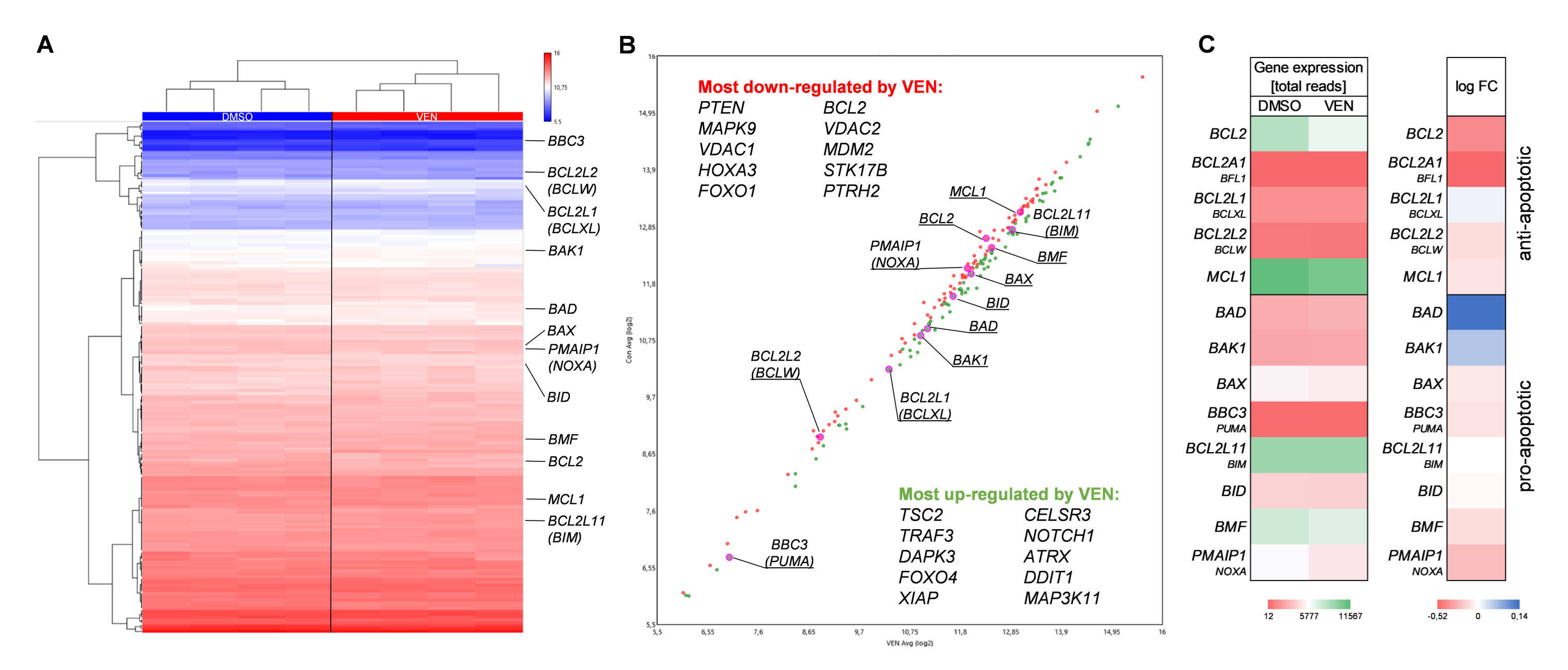

Supplement: Supplementary file 5 — Figure S3 [file 41420_2022_1093_MOESM5_ESM.png]

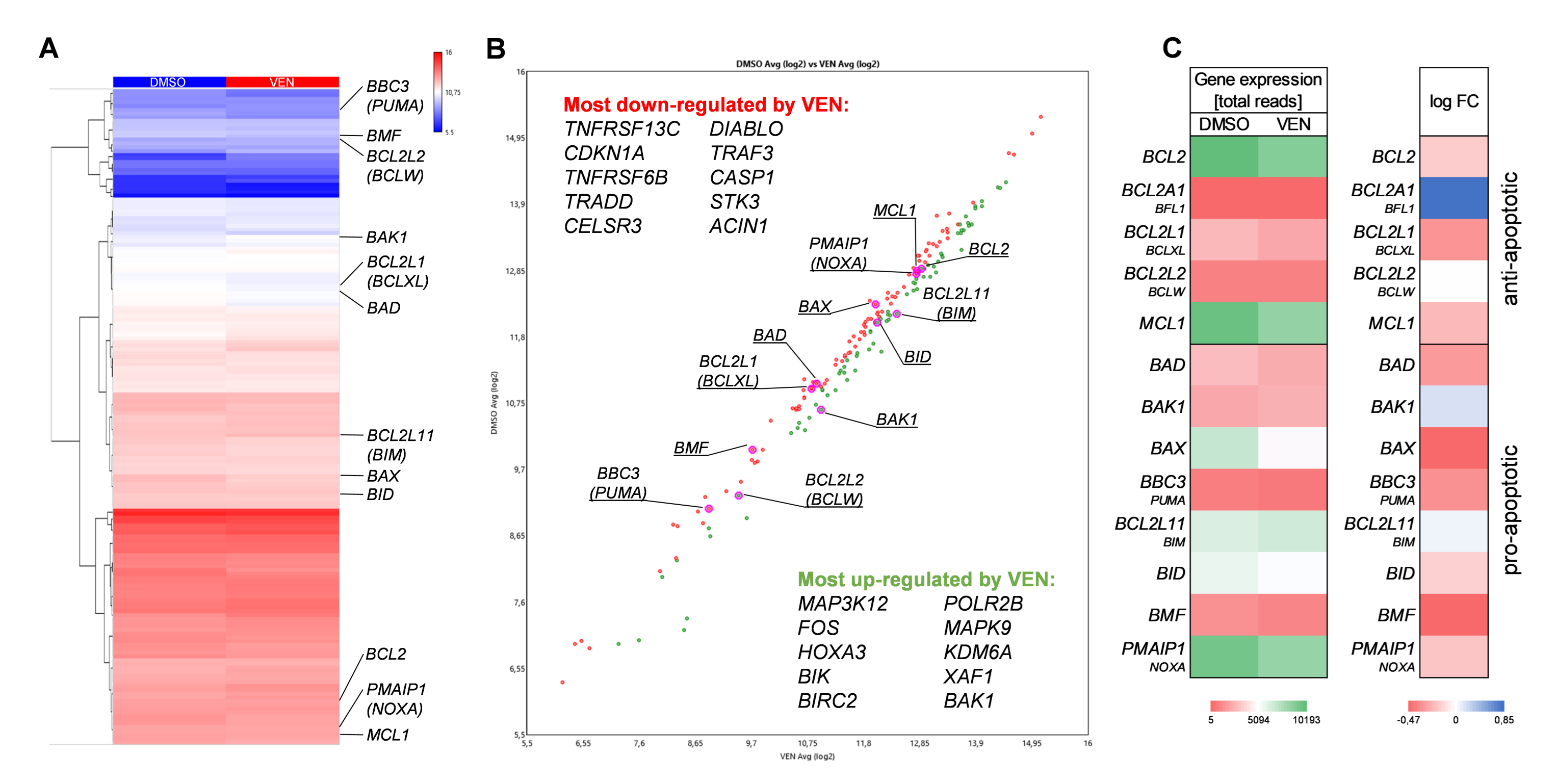

Supplement: Supplementary file 6 — Figure S4 [file 41420_2022_1093_MOESM6_ESM.png]

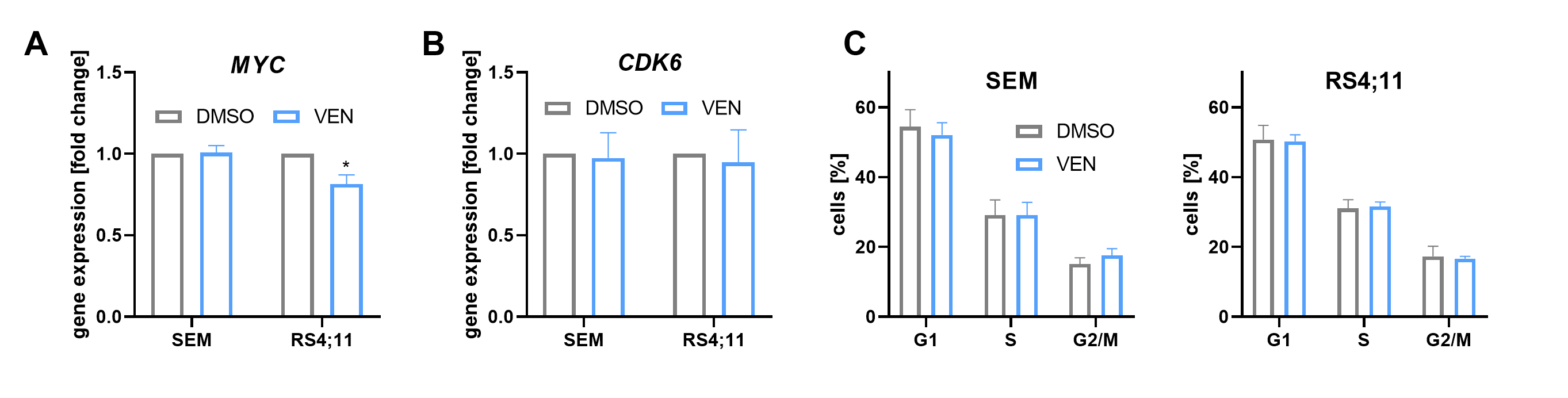

Supplement: Supplementary file 7 — Figure S5 [file 41420_2022_1093_MOESM7_ESM.png]

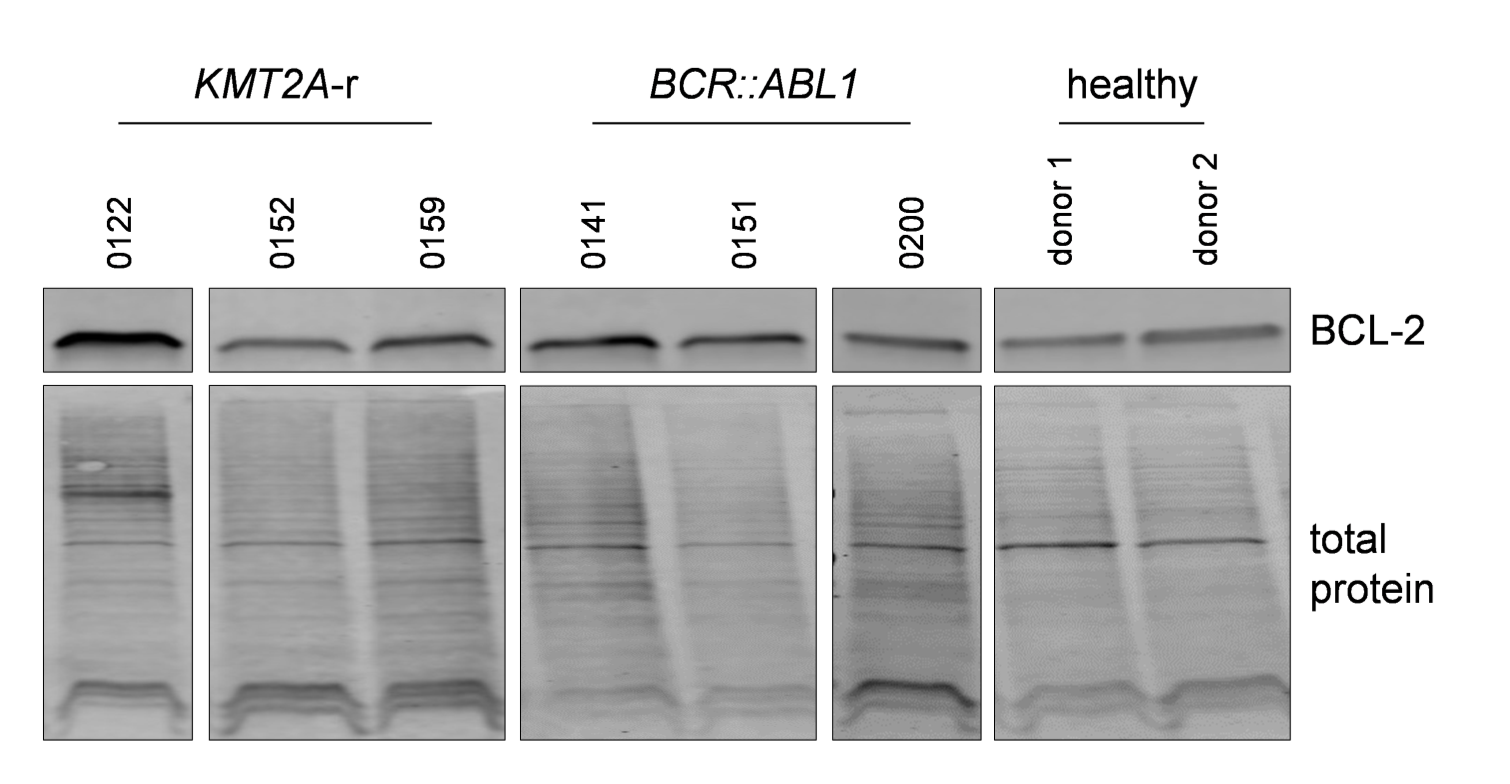

Supplement: Supplementary file 8 — Figure S6 [file 41420_2022_1093_MOESM8_ESM.png]

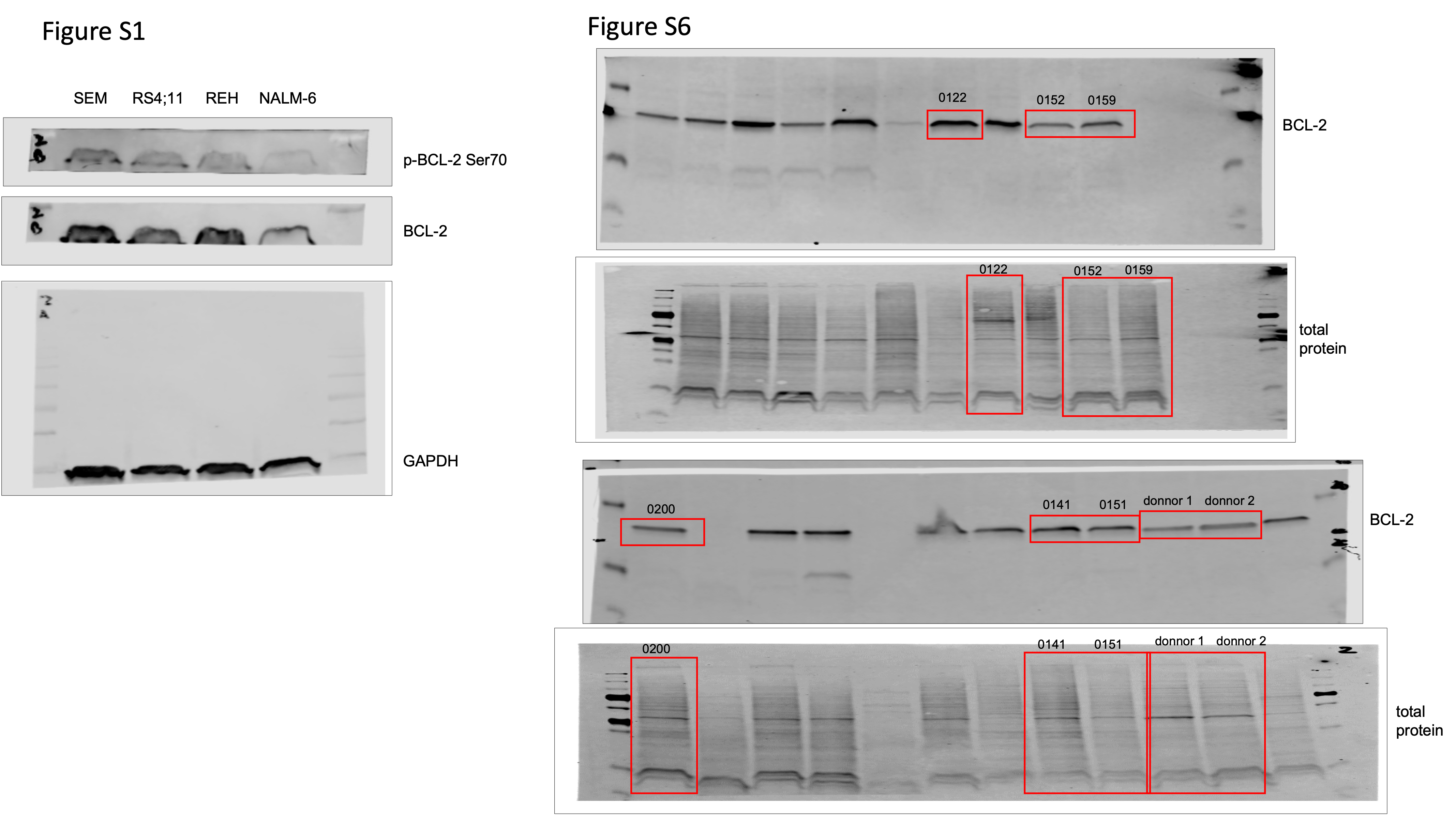

Supplement: Supplementary file 11 — Original Western Blots [file 41420_2022_1093_MOESM11_ESM.png]
