# Supplementary material for: Effective tumor cell abrogation via Venetoclax-mediated BCL-2 inhibition in KMT2A-rearranged acute B-lymphoblastic leukemia
Source: Cell Death Discov. 2022 Jul 1;8:302. doi: 10.1038/s41420-022-01093-3 (PMC9249764; doi:10.1038/s41420-022-01093-3)
Supplement: Supplementary file 9 — Supplementary Figure Legends [file 41420_2022_1093_MOESM9_ESM.docx]

**Effective tumor cell abrogation via Venetoclax-mediated BCL‑2 inhibition in *KMT2A*-rearranged acute B-lymphoblastic leukemia**

Anna Richter^1,^*, Sandra Lange^1^, Clemens Holz^1^, Luisa Brock^1^, Thomas Freitag^1^, Anett Sekora^1^, Gudrun Knuebel^1^, Saskia Krohn^1^, Rico Schwarz^2^, Burkhard Hinz^2^, Hugo Murua Escobar^1^, Christian Junghanss^1^

Supplementary Figure Legends

Figure S1: Basal protein expression of phosphorylated and total BCL‑2 in B‑ALL cell lines SEM, RS4;11, REH and NALM‑6. **A** Analyses were carried out by immunoblot using GAPDH as loading control. **B**Bands of total BCL‑2, phosphorylated BCL‑2 and GAPDH were quantified. Relative BCL‑2 signal intensity was calculated by normalization to GAPDH. **C** The BCL‑2 phosphorylated-to-total protein expression ratio was assessed using the relative signal intensities of p‑BCL‑2 and total BCL‑2 normalized to GAPDH bands.

Figure S2: Differential gene expression of 208 genes involved in apoptosis signaling in SEM and RS4;11 cells. **A**Principal component analysis of 4 (SEM) or 3 (RS4;11) biological replicates to assess the basal gene expression levels before VEN treatment. **B** Hierarchically clustered heatmap of SEM and RS4;11 replicate log2 expression values. Red and blue color indicates high and low read counts, respectively. **C**Scatter plot demonstrating aberrantly expressed genes in SEM and RS4;11. Genes marked in red and green exhibit higher expression values in RS4;11 and SEM, respectively. Mean log2 expression values. **D** Volcano plot of all analyzed genes. Genes with higher expression in SEM cells are located on the left-hand side while genes up-regulated in RS4;11 are distributed on the right. Genes involved in BCL‑2 signaling are highlighted (green, higher expression in SEM; red, higher expression in RS4;11). **E** Detailed analysis of absolute gene expression values (left panel) and log fold change (log FC, right panel) values following VEN exposure in 13 BCL‑2 pathway genes. Total read and log FC values were calculated from mean expression values of 4 (SEM) or 1 (RS4;11) biological replicates. For gene expression total read counts, red and green color indicates low and high read counts, respectively. For log FC values, red and green color symbolizes down- and up-regulation following VEN incubation, respectively.

Figure S3: Effect of 48 h VEN incubation (10 nM) on apoptosis and leukemia-related pathway gene expression levels in SEM cells with a focus on the 13 BCL‑2 pathway genes. **A,B**Genes were filtered by average total read count and only 176 genes with at least 50 reads per sample were included to justify biological and statistical relevance of observed changes. **A** Hierarchically clustered heatmap of log2 expression values. Red and blue color indicates high and low read counts, respectively. **B** Scatter plot demonstrating changes in gene expression following VEN exposure. Average log2 expression values. Red and green dots symbolize down- and up-regulated genes following VEN treatment, respectively. **C** Detailed analysis of absolute gene expression values (left panel) and log fold change (log FC, right panel) values following VEN exposure. Total read and log FC values were calculated from mean expression values. For gene expression total read counts, red and green color indicates low and high read counts, respectively. For log FC values, red and green color symbolizes down- and up-regulation following VEN incubation, respectively.

Figure S4: Effect of 48 h VEN incubation (2.5 nM) on apoptosis and leukemia-related pathway gene expression levels in RS4;11 cells with a focus on the 13 BCL‑2 pathway genes. **A,B**Genes were filtered by average total read count and only 175 genes with at least 50 reads per sample were included to justify biological and statistical relevance of observed changes. **A** Hierarchically clustered heatmap of log2 expression values. Red and blue color indicates high and low read counts, respectively. **B** Scatter plot demonstrating changes in gene expression following VEN exposure. Average log2 expression values. Red and green dots symbolize down- and up-regulated genes following VEN treatment, respectively. **C** Detailed analysis of absolute gene expression values (left panel) and log fold change (log FC, right panel) values following VEN exposure. For gene expression total read counts, red and green color indicates low and high read counts, respectively. For log FC values, red and green color symbolizes down- and up-regulation following VEN incubation, respectively.

Figure S5: Effect of VEN on *MYC* and *CDK6* gene expression and cell cycle phases. Cells were incubated with 10 nM (SEM) or 2.5 nM (RS4;11) for 72 h **A,B** Analysis of gene expression of *MYC* (**A**) and *CDK6* (**B**) in technical triplicates. Mean fold change ± SD of 4 (*MYC*) or 3 (*CDK6*) independent biological replicates; paired t test using dCT values. **C**  Flow cytometric analysis of cell cycle phases after fixation and PI staining. Mean ± SD of 3 biological replicates; paired t test.

Figure S6: Basal BCL‑2 protein expression of B‑ALL primary samples (*KMT2A*-rearranged (‑r): samples 0122, 0152, 0159; *BCR::ABL1* translocation: samples 0141, 0151, 0200) and healthy PBMCs. Analyses were carried out by immunoblot using a total protein stain as loading control.
