# Supplementary material for: Effective tumor cell abrogation via Venetoclax-mediated BCL-2 inhibition in KMT2A-rearranged acute B-lymphoblastic leukemia
Source: Cell Death Discov. 2022 Jul 1;8:302. doi: 10.1038/s41420-022-01093-3 (PMC9249764; doi:10.1038/s41420-022-01093-3)
Supplement: Supplementary file 10 — Supplementary methods [file 41420_2022_1093_MOESM10_ESM.docx]

**Effective tumor cell abrogation via Venetoclax-mediated BCL‑2 inhibition in *KMT2A*-rearranged acute B-lymphoblastic leukemia**

Anna Richter, Sandra Lange, Clemens Holz, Luisa Brock, Thomas Freitag, Anett Sekora, Gudrun Knuebel, Saskia Krohn, Rico Schwarz, Burkhard Hinz, Hugo Murua Escobar, Christian Junghanss

Supplementary Materials and Methods

**Cell viability and vitality assays**

Cell proliferation was assessed by trypan blue dye exclusion and manual counting of viable cells and metabolic activity was evaluated by WST—1 assay (Roche, Mannheim, Germany) after 72 h incubation with increasing concentrations (0.01 nM — 10 µM) of VEN (Hycultec, Beutelsbach, Germany) in three technical replicates. To identify toxicity in healthy blood cells, PBMCs of five healthy voluntary donors were isolated from whole blood using density gradient centrifugation (PAN—coll, PAN—biotech). For technical triplicates, 5 x 10^4^ cells per well were seeded into a 96‑well plate and incubated with 10 nM VEN or DMSO (negative control) for 24 h. Afterwards, 50 µl Calcein AM working solution (Sigma-Aldrich, St. Louis, MO, USA; final concentration 0.5 µM) per well were added and incubated for 30 min before detection using the Glomax plate reader (Promega, Madison, WI, USA, excitation 485 nm, emission 535 nm). To analyze potential hemolysis, 20 µl full blood was distributed into a 96‑well plate in technical triplicates and incubated with 10 nM VEN or 1 % SDS (Merck, Darmstadt, Germany, positive control) for 120 min. Absorption of the cell-free supernatant was determined at 540 nm (reference 690 nm).

**Morphology analyses**

Cytospins of cells incubated with 10 nM (SEM) or 2.5 nM (RS4;11) VEN or DMSO for 48 h were prepared by spinning 5 x 10^4^ cells onto microscopic slides using a Cytospin 3 centrifuge (Shandon, Frankfurt/Main, Germany, 10 min, 700 rpm). Slides were air-dried and for Pappenheim staining first incubated in May-Grünwald solution (Merck), then washed in buffer solution according to Weise (pH 7.2, Sigma-Aldrich), stained in Giemsa solution (Merck) for 20 min, and finally washed with buffer according to Weise followed by A. dest. Images were captured using the EVOS xl core microscope at 100—fold magnification.

**Flow cytometric apoptosis analysis**

Cells were incubated with increasing concentrations (0.01 nM — 10 µM) of VEN or DMSO (control) for 72 h, washed twice in PBS, resuspended in binding buffer (BD, Heidelberg, Germany) and subsequently stained with annexin V—FITC (BD) for 15 min. Propidium iodide (PI, 0.6 µg/ml) was added immediately prior to flow cytometric analysis using the FACSVerse (BD) with FACSuite software (BD). Annexin V^+^ PI^-^ cells were considered as early apoptotic and annexin V^+^ PI^+^ cells as late apoptotic or necrotic. Annexin V^-^/PI^-^ cells were designated as vital.

**Flow cytometric measurement of intracellular protein expression**

Cells were incubated with 10 nM (SEM) or 2.5 nM (RS4;11) VEN or DMSO (control) for 48 h, washed twice in cold PBS and fixed in 4 % formaldehyde solution (Polysciences Europe, Hirschberg an der Bergstraße, Germany) for 15 min. After PBS washing, cells were permeabilized in ice cold methanol for 30 min at room temperature followed by overnight incubation at ‑20°C. After two washing steps in PBS and incubation buffer (5 g/L bovine serum albumin (Serva, Heidelberg, Germany) in PBS), cells were blocked in 0.5 % bovine serum albumin solution for 10 min and subsequently stained with fluorophore-conjugated primary antibodies (BCL‑2‑PerCP‑Cy5.5 clone C‑2; p‑BCL‑2‑Alexa Fluor‑647 clone A‑11; MCL‑1‑PE clone 22; BCL‑XL‑PerCP‑Cy5.5 clone H‑5; Bax‑PE clone 2D2, all Santa Cruz Biotechnology (Dallas, TX, USA); Cleaved Caspase‑3‑Alexa Fluor‑488 polyclonal (Cell Signaling, Danvers, MA, USA)) for 1 h at room temperature. Stained cells were washed twice in incubation buffer, collected in 300 µl PBS and analyzed using the FACSVerse (BD) device with FACSuite software (BD).

**Flow cytometric cell cycle analysis**

Cells were incubated with 10 nM (SEM) or 2.5 nM (RS4;11) VEN for 72 h, harvested and washed twice in PBS before fixation in ice-cold 70 % ethanol for 24 h at ‑20°C. Cells were again washed in PBS and incubated with 1 mg/ml ribonuclease A (Sigma-Aldrich) at 37°C for 45 min before further washing. Finally, cells were stained with 50 µg/ml PI and analyzed by flow cytometry (FACSVerse (BD) with FACSuite software (BD)).

**Immunoblot**

Immunoblotting was performed as previously described ^1^. The GAPDH antibody was obtained from Invitrogen (Waltham, MA, USA) and diluted 1:20 000 in 1:5 blocking buffer (LI‑COR, Lincoln, NE, USA)/PBS. All other primary antibodies were purchased from Cell Signaling and diluted 1:1 000. IRDye® secondary antibodies were obtained from LI‑COR and diluted 1:5 000. Blots were processed and cropped using Image Studio Lite 5.2 software and MS PowerPoint (2016) to improve clarity and conciseness. Original blots are uploaded as a separate file. For quantification, band intensities were assessed using Image Studio Lite 5.2 software. To determine the p‑BCL‑2/BCL‑2 ratio, the total protein expression was measured using Revert™ Total Protein Stain (LI‑COR). p‑BCL‑2 and BCL‑2 band intensities were normalized to the total protein expression of the respective lane and normalized protein expression values were used to calculate the phosphorylated to total BCL‑2 protein expression. For quantification of the expression of all other proteins, the individual bands were normalized to the respective total protein expression or GAPDH protein expression.

**Bax translocation assay using immunofluorescence imaging**

SEM and RS4;11 cells were incubated with vehicle or 10 nM or 2.5 nM VEN, respectively, for 48 h before cells were harvested and washed twice in cold PBS. Cytospins were prepared, stained and analyzed as previously described ^1^. Images were taken using the Axio Observer inverted microscope (Zeiss, Oberkochen, Germany) and Eclipse TE200 camera (Nikon, Minato, Japan) and processed using ZEN Imaging software (Zeiss).

***MYC* and *CDK6* gene expression analysis**

SEM and RS4;11 cells were incubated with DMSO or 10 nM (SEM) or 2.5 nM VEN (RS4;11) for 72 h. Cells were harvested, washed twice in PBS and RNA isolation was performed using the RNeasy® Mini kit (Qiagen) followed by cDNA synthesis using random hexamer primers, 5x RT buffer complete, dNTP mix and Reverase™ (all Bioron, Römerberg, Germany) according to the manufacturer’s protocol. Gene expression was measured using 25 ng cDNA with SensiFAST™ Probe Lo‑ROX master mix (Bioline, London, UK) and TaqMan probes Hs00153408_m1 (*MYC*) and Hs01026371_m1 (*CDK6*) (all Thermo Fisher Scientific) in a ViiA 7 Real‑Time PCR System (Thermo Fisher Scientific) in technical triplicates. *GAPDH* primers were designed manually and as follows: forward TCACCAGGGCTGCTTTTAAC; reverse GGGTGGAATCATATTGGAACA; quencher TGCCATCAATGACCCCTTCATTG. Reactions were performed at 50°C for 2 min, 95°C for 10 min and followed by 40 cycles of 15 s at 95°C and 1 min at 60°C. All mean C_T_ values were normalized to the respective *GAPDH* mean C_T_ and changes in gene expression following VEN incubation were calculated using the 2^-ΔΔCT^ formula.

**Tumor cell isolation and subsequent analyses**

For bone marrow cell isolation, femurs and tibiae were collected in PBS and processed as described in ^2^. Spleens were first weighed and measured and subsequently stored in PBS until being passed through a 100 µm cell strainer (Greiner Bio-One, Kremsmünster, Austria). Erythrocyte lysis of spleen cells, and bone marrow cells when pellets were red, was performed using cold erythrocyte lysis buffer (10 mM KHCO_3_, 155 mM NH_4_Cl, 0.1 mM EDTA) for  7 min. Cells were washed twice in cold PBS and cell numbers were determined. 5 x 10^5^ – 1 x 10^6^ cells were used for flow cytometric determination of tumor cell frequencies in bone marrow, spleen and PB as described above (GFP^+^ or CD45^+^/CD19^+^). 2 x 10^5^ cells were dissolved in 800 µl PBS for cytospin preparations and subsequent morphological analyses as described in the morphology section above.

**Pharmacokinetic analyses**

To estimate the maximum VEN concentration and duration in VEN availability in PB, full blood samples from control animals (n=5) and VEN-treated mice (n=3‑8) was collected 1 h, 2 h, 24 h and 72 h following VEN or vehicle p.o. application. Samples were 1:3 diluted in ultragradient HPLC grade water (Merck) to achieve hemolysis and centrifuged at 26 342 x g for 5 min. A standard curve was performed using hemolyzed blood of untreated NSG mice retrospectively spiked with known VEN concentrations. 20 µl of standard or sample were then mixed with 20 µl H_2_O and 10 µl acridine orange (0.5 µM, final concentration 0.1 µM). 800 µl ethyl acetate was added for extraction of compounds. Samples were vortexed and centrifuged at 26 342 x g for 5 min. 700 µl of the sample were subsequently evaporated to dryness using a SpeedVac SPD130DLX centrifuge (Thermo Fisher Scientific) and reconstituted in 100 µl of 50 % acetonitrile/H_2_O, of which 10 µl were injected for quantification. Mass spectrometric analysis was carried out using a Prominence LC‑20AD HPLC system (Shimadzu, Duisburg, Germany) coupled to a LCMS‑8050 triple quadrupole mass spectrometer (Shimadzu). Separation was performed on a Multospher® 120 RP 18 AQ‑5µ (125 x 2 mm, 5 µm particle size; CS-Chromatographie Service GmbH, Langerwehe, Germany) equipped with a guard column. Water was chosen as solvent A and acetonitrile as solvent B, with both containing 0.2 % formic acid. The gradient was started with 0 % of B and was increased to 100 % of B over 5 min. Subsequently, the gradient was immediately changed back to 0 % of B and the system was re-equilibrated for 3 min. The flow rate was 0.5 ml/min with an autosampler temperature of 15°C and oven temperature of 60°C. The calibration curve ranged from 0.01 µM to 1 µM (0.01; 0.025; 0.05; 0.1; 0.25; 0.5 and 1 µM) and a linear regression was performed for quantification. VEN and the internal standard acridine orange were ionized by electrospray ionization in positive mode. Multiple reaction monitoring was used to identify and quantify the analytes. The specific *m/z* transitions are shown in the table below. Specific mass spectrometer parameters were nebulization gas flow 3.0 L/min, interface voltage 4.0 kV, interface temperature 300°C, heating and drying gas flow 10.0 L/min, temperature of the desolvation line 250°C and desolvation temperature 526°C. The LabSolutions software from Shimadzu (version 5.97, Shimadzu, Duisburg, Germany) was used for the evaluation.

Parameters and transitions for mass spectrometric analysis of VEN and acridine orange. Marked *m/z* transitions with * are used for quantification.

| Compound | Precursor  *m/z* | Product  *m/z* | Dwell time (ms) | Q1 Pre Bias (V) | Collision  energy (V) | Q3 Pre Bias (V) |
| --- | --- | --- | --- | --- | --- | --- |
| Venetoclax | 869.3 | 637.15 321.15 | 91  91 | -26.0  -34.0 | -31.0  -40.0 | -26.0  -17.0 |
| Venetoclax | 434.8 | 321.15 *  233.0  177.05 | 59  59  59 | -13.0  -13.0  -13.0 | -26.0  -13.0  -20.0 | -24.0  -26.0  -19.0 |
| Acridine orange | 266.3 | 250.1 *  234.0  222.1 | 59  59  59 | -14.0  -14.0  -14.0 | -35.0  -52.0  -34.0 | -28.0  -26.0  -24.0 |

**Doubling time calculation**

Doubling times were calculated using the following formula with t_1_ and t_2_ indicating the time points (days) since tumor cell injection used for doubling time calculations. PB blast frequencies were assessed at d14 and d21 to estimate the doubling time during treatment while d28/d35 or d35/d42 were used for post-treatment values in SEM- and RS4;11-derived models, respectively.

$$Doubling time=\frac{\left( t_{2}- t_{1} \right)*\log\left( 2 \right)}{log(blast frequency{(t}_{2}))- log(blast frequency(t_{1}))}$$

References

1 Richter A, Fischer E, Holz C, Schulze J, Lange S, Sekora A *et al.* Combined Application of Pan-AKT Inhibitor MK-2206 and BCL-2 Antagonist Venetoclax in B-Cell Precursor Acute Lymphoblastic Leukemia. *Int J Mol Sci* 2021; **22**: 2771.

2 Amend SR, Valkenburg KC, Pienta KJ. Murine hind limb long bone dissection and bone marrow isolation. *J Vis Exp* 2016; **2016**: 3–6.
